# Effects of delayed-release dimethyl fumarate on MRI measures in the Phase 3 DEFINE study

**DOI:** 10.1007/s00415-014-7412-x

**Published:** 2014-07-03

**Authors:** Douglas L. Arnold, Ralf Gold, Ludwig Kappos, Amit Bar-Or, Gavin Giovannoni, Krzysztof Selmaj, Minhua Yang, Ray Zhang, Monica Stephan, Sarah I. Sheikh, Katherine T. Dawson

**Affiliations:** 1 NeuroRx Research, Montreal, QC Canada; 2 Montreal Neurological Institute and Hospital, McGill University, 3801 University Street, Suite 207, Montreal, QC H3A 2B4 Canada; 3 Department of Neurology, St Josef-Hospital/Ruhr-University Bochum, Bochum, Germany; 4 Departments of Neurology and Biomedicine, University Hospital Basel, Basel, Switzerland; 5 Blizard Institute, Barts and the London School of Medicine and Dentistry, Queen Mary University of London, London, UK; 6 Medical University of Lodz, Lodz, Poland; 7 Biogen Idec Inc., Cambridge, MA USA

**Keywords:** Brain atrophy, Delayed-release dimethyl fumarate, Lesion, MRI, Relapsing–remitting multiple sclerosis

## Abstract

In the Phase 3 DEFINE study, delayed-release dimethyl fumarate (DMF) 240 mg twice (BID) and three times daily (TID) significantly reduced the mean number of new or enlarging T2-hyperintense lesions and gadolinium-enhancing (Gd+) lesion activity at 2 years in patients (MRI cohort; *n* = 540) with relapsing–remitting MS. The analyses described here expand on these results by considering additional MRI measures (number of T1-hypointense lesions; volume of T2-hyperintense, Gd+, and T1-hypointense lesions; brain atrophy), delineating the time course of the effects, and examining the generality of the effects across a diverse patient population. Reductions in lesion counts with delayed-release DMF BID and TID, respectively, vs. placebo were apparent by the first MRI assessment at 6 months [T2-hyperintense: 80 and 69 % reduction (both *P* < 0.0001); Gd+, 94 and 81 % reduction (both *P* < 0.0001); T1-hypointense: 58 % (*P* < 0.0001) and 48 % (*P* = 0.0005) reduction] and maintained at 1 and 2 years. Reductions in lesion volume were statistically significant beginning at 6 months for T2-hyperintense [*P* = 0.0002 (BID) and *P* = 0.0035 (TID)] and Gd+ lesions [*P* = 0.0059 (BID) and *P* = 0.0176 (TID)] and beginning at 1 year [*P* = 0.0126 (BID)] to 2 years [*P* = 0.0063 (TID)] for T1-hypointense lesions. Relative reductions in brain atrophy from baseline to 2 years (21 % reduction; *P* = 0.0449) and 6 months to 2 years (30 % reduction; *P* = 0.0214) were statistically significant for delayed-release DMF BID. The effect of delayed-release DMF on mean number of new or enlarging T2-hyperintense lesions and Gd+ lesion activity was consistent across pre-specified patient subpopulations. Collectively, these results suggest that delayed-release DMF favorably affects multiple aspects of MS pathophysiology.

**Electronic supplementary material:**

The online version of this article (doi:10.1007/s00415-014-7412-x) contains supplementary material, which is available to authorized users.

## Introduction

MRI is an important tool for monitoring MS disease activity and progression in both clinical practice and clinical trials [1, 2]. Gadolinium-enhancing (Gd+) lesion counts on T1-weighted images, and new or enlarging T2-hyperintense lesions, give an indication of recent inflammation and may predict relapse rate in the short term [2, 3]. T2-weighted images allow a quantitation of the accumulated extent, or overall burden, of focal white matter disease [1, 4], whereas chronic (unenhancing) T1-hypointense lesions have been more strongly associated with tissue destruction and axonal loss, and their persistence is associated with increased disability and permanent neurological deficit [5, 6]. Overall reduction in brain volume over time is considered to be a marker of neurodegeneration, and shows a relatively strong correlation with physical disability and cognitive impairment [7]. A goal of MS treatment is to attenuate or delay these changes on MRI by counteracting the inflammatory neurodegeneration and demyelination that define the disease.

Delayed-release dimethyl fumarate (DMF; known as Tecfidera in countries in which it is approved and referred to as BG-12 during clinical development) is a novel oral therapeutic tested in people with relapsing–remitting MS (RRMS). Preclinical studies indicate that delayed-release DMF exerts anti-inflammatory and neuroprotective effects via activation of the nuclear factor (erythroid-derived 2)-like2 (Nrf2) pathway [8, 9] and also via Nrf2-independent mechanisms [10, 11]. In the randomized, placebo-controlled, Phase 3 DEFINE trial, delayed-release DMF 240 mg twice (BID) and three times daily (TID) demonstrated efficacy on clinical endpoints, including annualized relapse rate, risk of relapse, and risk of confirmed disability progression, combined with an acceptable safety profile, over 2 years [12]. Delayed-release DMF also demonstrated significant efficacy on MRI endpoints including mean number of new or enlarging T2-hyperintense lesions (BID: 85 % reduction; TID: 74 % reduction; both *P* < 0.001 vs. placebo) and Gd+ lesion activity (BID: 90 % reduction; TID: 73 % reduction; both *P* < 0.001 vs. placebo), at 2 years [12]. These findings were supported by the results of a second Phase 3 trial, the CONFIRM study, which additionally evaluated subcutaneous glatiramer acetate as an active reference treatment (rater-blinded) [13].

The present analyses were conducted to expand on the previously reported MRI results from the DEFINE study by considering additional MRI measures, delineating the time course of the effects, and examining the generality of the effects across a diverse patient population. Here, we report the effects of delayed-release DMF on the number of new or enlarging T2-hyperintense lesions and number of Gd+ lesions at 6 months, 1 and 2 years; the number of new non-enhancing T1-hypointense lesions at 6 months, 1 and 2 years; the volume of all T2-hyperintense, Gd+, and T1-hypointense lesions at 6 months, 1 and 2 years; brain atrophy at 2 years; and the number of T2-hyperintense and Gd+ lesions at 2 years in subgroups of patients stratified by baseline demographic and disease characteristics.

## Methods

### Standard protocol approvals, registrations, and patient consents

The DEFINE study (ClinicalTrials.gov identifier NCT00420212) was approved by central and local ethics committees and conducted in accordance with the International Conference on Harmonisation Guidelines for Good Clinical Practice [14] and the Declaration of Helsinki [15]. All patients were fully informed of approved MS therapies as an alternative to participation in a placebo-controlled trial, and provided written informed consent and re-consent after confirmed relapse or disability progression (after discussion of treatment options as detailed below).

### Patients

As reported previously [12], eligible patients were aged 18–55 years, with a diagnosis of RRMS, a baseline score of 0–5.0 on the Expanded Disability Status Scale (EDSS), and disease activity defined by at least one clinically documented relapse within 12 months before randomization, or a brain MRI scan with at least one Gd+ lesion within 6 weeks before randomization. Key exclusion criteria included progressive forms of MS, other major disease that would preclude participation in a clinical trial, pre-specified abnormal laboratory results, or recent exposure to contraindicated medications [12].

### Study design

Patients were randomized 1:1:1 to receive double-blind treatment with oral delayed-release DMF 240 mg BID or TID or placebo [12]. Randomization was centralized and stratified by site. Each study center used separate examining and treating neurologists who were blinded throughout. The examining neurologist conducted neurological assessments while the treating neurologist was responsible for all aspects of patient care, including the treatment of relapses and other disease symptoms. Patients were eligible to switch to an alternative MS therapy if they had completed 48 weeks of blinded treatment and experienced ≥1 confirmed relapse after 6 months, or at any time if they had experienced confirmed disability progression.

### Study procedures and endpoints

As reported elsewhere, the primary endpoint was the proportion of patients who had experienced an MS relapse by 2 years [12]. Secondary MRI endpoints at 2 years included the number of new or enlarging T2-hyperintense lesions and the number of Gd+ lesions on brain MRI. Tertiary endpoints included number of new or enlarging T2-hyperintense lesions and number of Gd+ lesions at 1 year; number of new T1-hypointense lesions at 1 and 2 years; volume of all T2-hyperintense, Gd+, and T1-hypointense lesions at 1 and 2 years; and brain atrophy at 2 years.

### MRI

Brain MRI scans with and without gadolinium were performed in patients from a subset of sites with full MRI capabilities using a standardized acquisition supervised by the MRI reading center (NeuroRx Research, Montreal, QC, Canada). Approximately 95 % of patients at these sites chose to participate. MRI scans were obtained at baseline (or any time between screening and baseline) and 6 months, 1 and 2 years and were not to be performed within 30 days of a course of steroids. MRI scans provided full head coverage and included the following sequences: PD- and T2-weighted 2D multislice turbo/fast spin-echo, T1-weighted 3D spoiled gradient-recalled echo (pre-contrast), 2D T2-weighted FLAIR, and 3D spoiled gradient-recalled echo T1-weighted (post-contrast).

All original digital data for all MRI images were transferred from each of the sites to the MRI reading center for blinded evaluation. Lesion-based measurements performed included: T2-weighted lesion count (at baseline); new or enlarging T2-weighted lesion count (all post-baseline visits); T2-weighted lesion volume (all MRI visits); Gd+ lesion count and volume (all MRI visits); T1-weighted hypointense (non-enhancing) lesion count at baseline; new T1-weighted non-enhancing lesion count (all post-baseline visits); and total volume of non-enhancing T1-weighted lesions (all MRI visits). T2-weighted lesions were segmented using locally developed software [16] and manually corrected as necessary. Lesions had to be at least three voxels in size to be counted. T1 lesions were segmented within T2-weighted lesions using a threshold of 85 % of the intensity of surrounding normal-appearing white matter (which corresponded approximately to the intensity of grey matter). Regions of acute T1 hypointensity associated with gadolinium enhancement were removed. Baseline lesions had to be surrounded by normal-appearing tissue. New lesion counts were made relative to the prior visit and added over time intervals as appropriate to express results relative to baseline. For assessment of brain atrophy, normalized brain volume was determined at baseline, and percentage brain volume change (PBVC) calculated automatically for each post-baseline MRI visit relative to baseline.

### Statistical analyses

The MRI cohort comprised patients who consented to participate in MRI analysis and had any MRI data, and was a subset of the overall study intention-to-treat (ITT) population (randomized patients who received at least one dose of study treatment).

The numbers of new or enlarging T2-hyperintense and new non-enhancing T1-hypointense lesions were analyzed using a negative binomial regression model; the number of Gd+ lesions was analyzed using an ordinal logistic regression model; and brain atrophy was analyzed using percentage changes compared between treatment groups using an analysis of covariance on ranked data. The analytical models included adjustments for region (defined by type of healthcare system and access to healthcare, in addition to geography) and baseline characteristics, including number or volume of baseline lesions, as appropriate.

For brain atrophy analyses, the pre-specified analysis of interest was the PBVC from 6 months to 2 years. Six months was chosen as the baseline reference time point because in RRMS studies of therapies with anti-inflammatory properties, a greater relative decrease in brain volume has been observed in the therapeutic agent arm within the first several months of treatment, presumably due to a greater reduction in inflammation and edema [17]. Adjustments were made for region and normalized brain volume at the reference time point.

A sequential (closed) testing procedure was used to control the overall type I error rate due to multiple comparisons for primary and secondary endpoints; a similar procedure was not applied to tertiary endpoints. Formal testing of delayed-release DMF BID vs. placebo groups was only undertaken if the analysis of delayed-release DMF TID vs. placebo groups was statistically significant (*P* ≤ 0.05). Data obtained after patients switched to alternative MS medication were excluded. MRI lesion count and PBVC endpoint analyses used all pre-switch data and then imputed data after withdrawal or switching to a rescue medication using a constant rate assumption. For MRI volume data, missing data were imputed using the mean of each treatment group at each visit.

## Results

### Patients

**Fig. 1:**
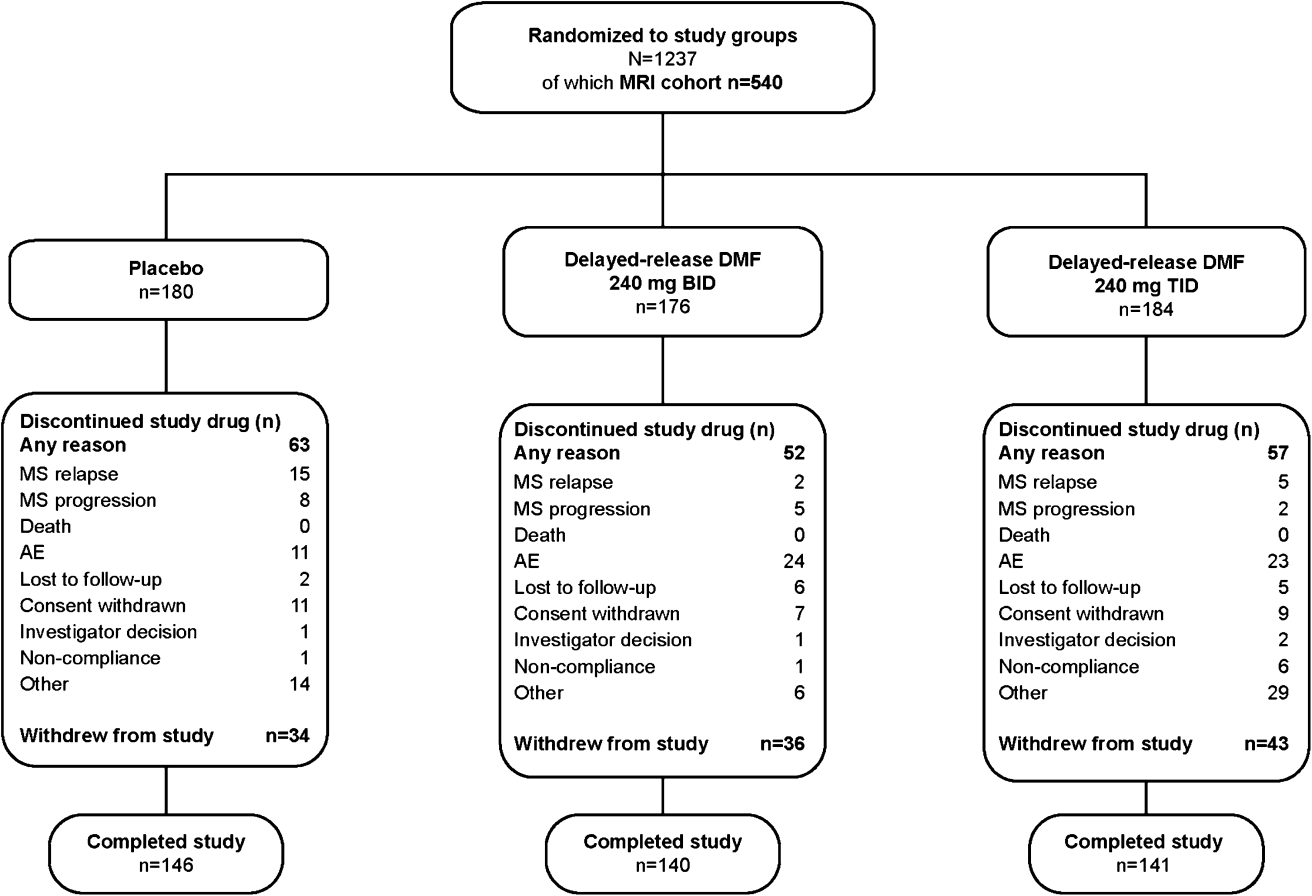
Patient disposition: MRI cohort. *BID* twice daily, *MS* multiple sclerosis, *TID* three times daily

The disposition and baseline characteristics of the overall ITT population (*n* = 1,234) have been reported previously [12]. The MRI cohort comprised 540 patients in the ITT population who were enrolled at sites that participated in the MRI portion of the study (76 of 198 sites in 14 countries worldwide) and who had any MRI data: 180, 176, and 184 patients in the placebo, delayed-release DMF BID, and delayed-release DMF TID groups, respectively (Fig. 1). The MRI cohort displayed baseline characteristics that were similar to the non-MRI cohort (Table e-1) and the overall DEFINE ITT population [12], and were broadly comparable across treatment groups (Table 1). In both the MRI cohort and overall ITT population, 41 % of patients had received prior approved disease-modifying therapy. The mean time on study was 87.8, 85.8, and 82.2 weeks in the placebo, delayed-release DMF BID, and delayed-release DMF TID groups of the MRI cohort, respectively.

**Table 1:** Demographics and baseline characteristics of the MRI cohort

Characteristic	Placebo (*n* = 180)	Delayed-release DMF BID (*n* = 176)	Delayed-release DMF TID (*n* = 184)
Age, mean (SD) years	38.3 (9.16)	38.3 (9.31)	38.5 (8.59)
<40 years, *n* (%)	90 (50)	92 (52)	101 (55)
≥40 years, *n* (%)	90 (50)	84 (48)	83 (45)
Female, *n* (%)	141 (78)	136 (77)	140 (76)
McDonald criteria for diagnosis			
Criterion 1, *n* (%)	152 (84)	149 (85)	147 (80)
Criteria 2–4, *n* (%)	28 (16)	27 (15)	37 (20)
Previous approved disease-modifying therapy, *n* (%)^a^	77 (43)	68 (39)	74 (40)
Time since diagnosis, mean (SD) years	6.0 (5.76)	5.6 (5.54)	5.0 (4.96)
Relapses in previous year, mean (SD)	1.3 (0.73)	1.3 (0.64)	1.3 (0.57)
≤1 relapse, *n* (%)	122 (68)	123 (70)	134 (73)
≥2 relapses, *n* (%)	58 (32)	53 (30)	50 (27)
EDSS score, mean (SD)^b^	2.53 (1.25)	2.29 (1.17)	2.30 (1.19)
EDSS ≤2, *n* (%)	88 (49)	101 (57)	105 (57)
EDSS >2, *n* (%)	92 (51)	75 (43)	79 (43)
Number of T2 lesions, mean (SD)	49.2 (38.6)	47.6 (34.7)	55.8 (44.3)
T2 lesion volume, mean (SD) mm^3^	6,524.9 (7,601.50)	8,463.8 (10,058.73)	9,014.5 (11,769.21)
Number of Gd+ lesions, mean (SD)	1.6 (3.45)	1.2 (3.30)	1.2 (4.10)
Patients with Gd+ lesions, *n* (%)			
0 lesions	103 (57)	117 (66)	124 (67)
1–4 lesions	55 (31)	47 (27)	49 (27)
5–8 lesions	13 (7)	5 (3)	7 (4)
≥9 lesions	9 (5)	6 (3)	4 (2)
Unknown	0	1 (<1)	0
Number of T1-hypointense lesions, mean (SD)	27.3 (28.47)	27.8 (29.66)	33.6 (34.74)
Normalized whole brain volume, mean (SD) cm^3^	1,586.7 (81.7)	1,573.5 (85.8)	1,565.5 (93.1)

*BID* twice daily, *EDSS* Expanded Disability Status Scale, *Gd+* gadolinium-enhancing, *TID* three times daily

^a^Data shown for approved disease-modifying treatments only: interferon β-1a (29 % of all patients in the MRI cohort), glatiramer acetate (18 %), interferon β-1b (12 %), and natalizumab (3 %). Patients may have received more than one prior disease-modifying treatment

^b^Scores on the EDSS range from 0 to 10, with higher scores indicating a greater degree of disability. Baseline score was >5.0 for one patient in the placebo group and one patient in the TID group

### Lesion number and volume over 2 years

#### Full MRI cohort

**Fig. 2:**
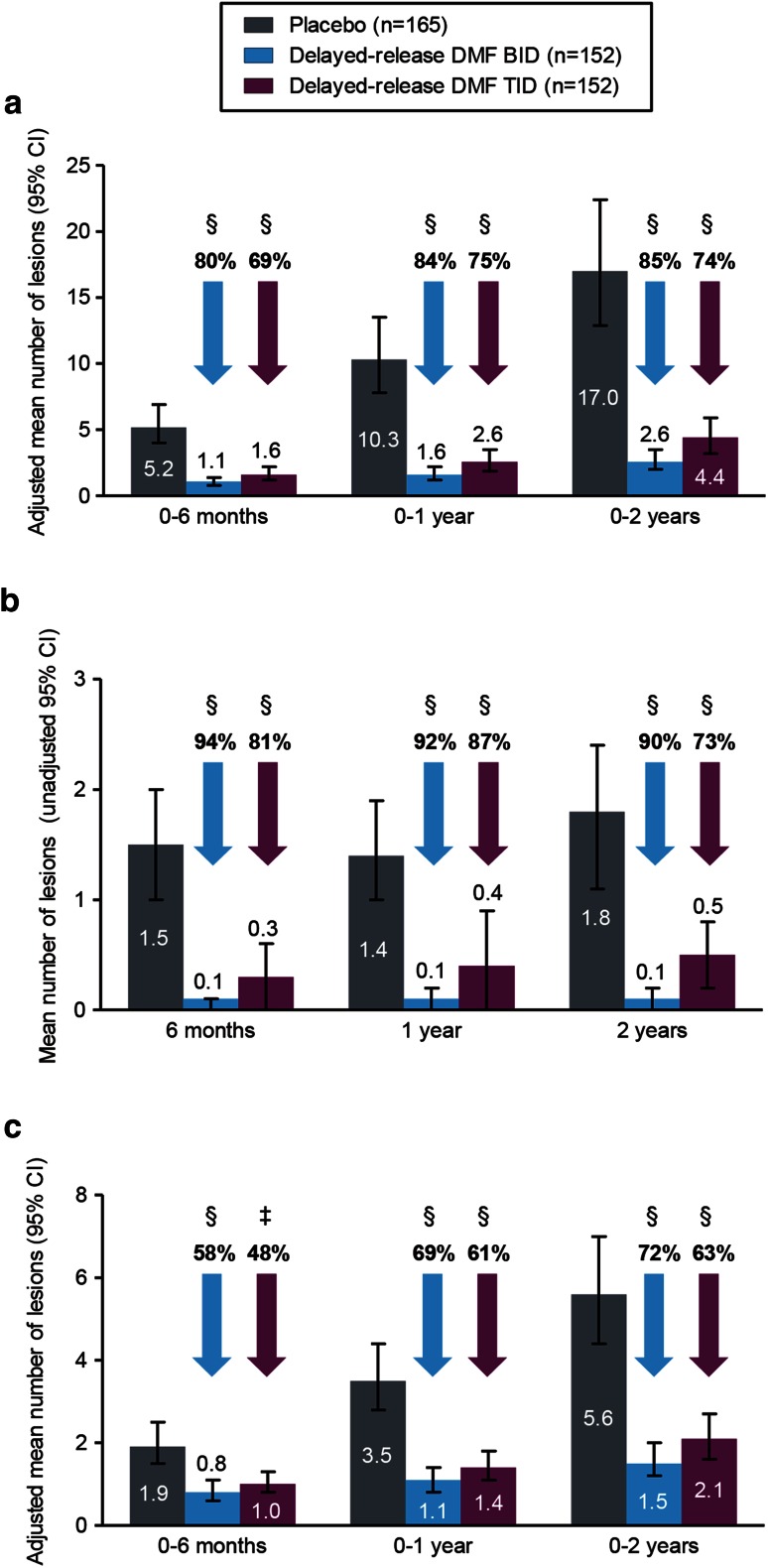
Mean number (±95 % CI) of new or enlarging T2-hyperintense lesions (**a**), Gd+ lesions (**b**), and new T1-hypointense lesions (**c**) at 6 months, 1 and 2 years. Patient numbers refer to those who provided data both at baseline and at each scheduled MRI analysis. Relative reductions (vs. placebo) in the risk of having a greater number of Gd+ lesions were based on the odds ratio from the pre-specified analysis model of ordinal logistic regression (a conservative method, chosen to minimize undue influence of outlier Gd+ lesion counts on the estimated treatment effect), for categories of patients with 0, 1, 2, 3–4, and ≥5 lesions. Comparisons vs. placebo were based on: (**a**) negative binomial regression, adjusted for region and baseline lesion volume; (**b**) ordinal regression, adjusted for region and baseline lesion number; (**c**) analysis of covariance on ranked data, adjusted for region and baseline lesion volume. *P < 0.05; **P < 0.01; ^‡^ *P* < 0.001; ^§^ *P* < 0.0001 vs. placebo. *BID* twice daily, *CI* confidence interval, *DMF* dimethyl fumarate, *Gd+* gadolinium-enhancing, *TID* three times daily

Compared with placebo, delayed-release DMF treatment at both doses resulted in statistically significant reductions in the number of brain lesions at the first MRI assessment on therapy (6 months), which were maintained to the end of the study. Delayed-release DMF reduced the mean number of new or enlarging T2-hyperintense lesions by 80 % (BID) and 69 % (TID) at 6 months, by 84 % (BID) and 75 % (TID) at 1 year, and by 85 % (BID) and 74 % (TID) at 2 years, compared with placebo (all *P* < 0.0001; Fig. 2a; Table e-2). Similar results in favor of delayed-release DMF were seen for Gd+ lesions (Fig. 2b; Table e-3), with relative odds reductions of 94 % (BID) and 81 % (TID) at 6 months, of 92 % (BID) and 87 % (TID) at 1 year, and of 90 % (BID) and 73 % (TID) at 2 years (all *P* < 0.0001). Delayed-release DMF reduced the mean number of new non-enhancing T1-hypointense lesions by 58 % (BID; *P* < 0.0001) and 48 % (TID; *P* = 0.0005) at 6 months, by 69 % (BID; *P* < 0.0001) and 61 % (TID; *P* < 0.0001) at 1 year, and by 72 % (BID; *P* < 0.0001) and 63 % (TID; *P* < 0.0001) at 2 years, compared with placebo (Fig. 2c; Table e-4). At study end, 93 % of patients in the BID group and 86 % in the TID group were free of Gd+ lesions, compared with 62 % in the placebo group (Table e-3).

**Fig. 3:**
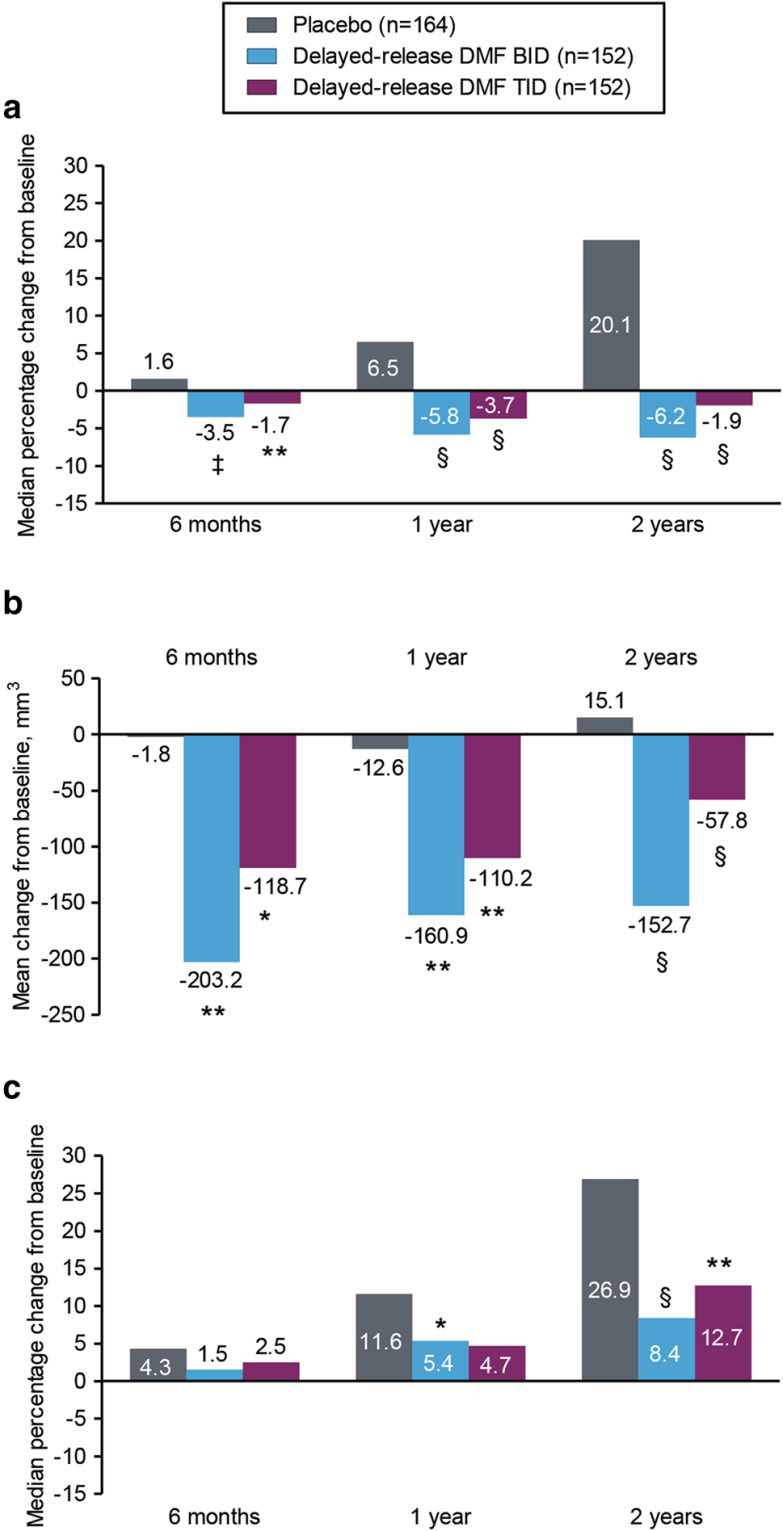
Median percentage change in volume of T2-hyperintense lesions (**a**), mean change from baseline in volume (mm^3^) of Gd+ lesions (**b**), and median percentage change in volume of T1-hypointense lesions (**c**) at 6 months, 1 and 2 years. Patient numbers refer to those who provided data both at baseline and at each scheduled MRI analysis. Comparisons vs. placebo were based on analysis of covariance on ranked data, adjusted for region and baseline lesion volume. *P < 0.05; **P < 0.01; ^‡^ *P* < 0.001; ^§^ *P* < 0.0001 vs. placebo. *BID* twice daily, *DMF* dimethyl fumarate, *Gd+* gadolinium-enhancing, *TID* three times daily

These reductions in lesion number were paralleled by reductions in lesion volume (Fig. 3a–c). Reductions in T2-hyperintense and Gd+ lesion volume were statistically significant beginning from 6 months onward. From baseline to 6 months, the median percentage change in T2-hyperintense lesion volume with delayed-release DMF BID and TID vs. placebo was −3.5 and −1.7 vs. +1.6 % (BID, *P* = 0.0002; TID, *P* = 0.0035); from baseline to 1 year, the median percentage change was −5.8 and −3.7 vs. +6.5 % (both *P* < 0.0001); and from baseline to 2 years, the median percentage change was −6.2 and −1.9 vs. +20.1 % (both *P* < 0.0001; Fig. 3a; Table e-2). At 6 months, the mean change in volume (mm^3^) of Gd+ lesions was −1.8, −203.2, and −118.7 for placebo, delayed-release DMF BID, and delayed-release DMF TID, respectively, which corresponds to a mean percentage change of −95.0 % (BID) and −82.0 % (TID) vs. +14.1 % (placebo; both *P* < 0.0001) (Fig. 3b; Table e-3). At 1 year, the mean change in volume (mm^3^) of Gd+ lesions was −12.6, −160.9, and −110.2 for placebo, delayed-release DMF BID, and delayed-release DMF TID, respectively, which corresponds to a mean percentage change of −88.5 % (BID) and −65.9 % (TID) vs. +97.4 % (placebo; both *P* < 0.0001) (Fig. 3b; Table e-3). At 2 years, the mean change in volume (mm^3^) of Gd+ lesions was +15.1, −152.7, and −57.8 for placebo, delayed-release DMF BID, and delayed-release DMF TID, respectively, which corresponds to a mean percentage change of −79.0 % (BID) and −52.6 % (TID) vs. +106.4 % (placebo; both *P* < 0.0001) (Fig. 3b; Table e-3). Reductions in T1-hypointense lesion volume were statistically significant beginning from 1 year onward (BID) and at 2 years (TID). From baseline to 6 months, the median percentage change in T1-hypointense lesion volume was +1.5 and +2.5 vs. +4.3 % (both *P* > 0.05); from baseline to 1 year, the median percentage change was +5.4 and +4.7 vs. +11.6 % (BID, *P* = 0.0126; TID, *P* > 0.05); and from baseline to 2 years, the median percentage change was +8.4 and +12.7 vs. +26.9 % (BID, *P* < 0.0001; TID, *P* = 0.0063; Fig. 3c; Table e-4).

The robustness of these findings was confirmed by sensitivity analyses, based on all observed data, and on observed data obtained prior to the start of alternative therapy in patients who switched MS therapy (Figure e-1).

#### Patient subpopulations

The effect of delayed-release DMF on the mean number of new or enlarging T2-hyperintense lesions and on the number of Gd+ lesions was consistent across a range of pre-specified patient subpopulations. In patients stratified by baseline characteristics (gender, age <40 vs. ≥40 years, ≤1 vs. ≥2 relapses in the year prior to study, McDonald criteria, prior MS treatment, EDSS score, T2-hyperintense lesion volume, and Gd+ lesion status), results showed consistent reductions in lesion numbers with either dose of delayed-release DMF compared with placebo (Figure e-2).

### Brain atrophy

**Fig. 4:**
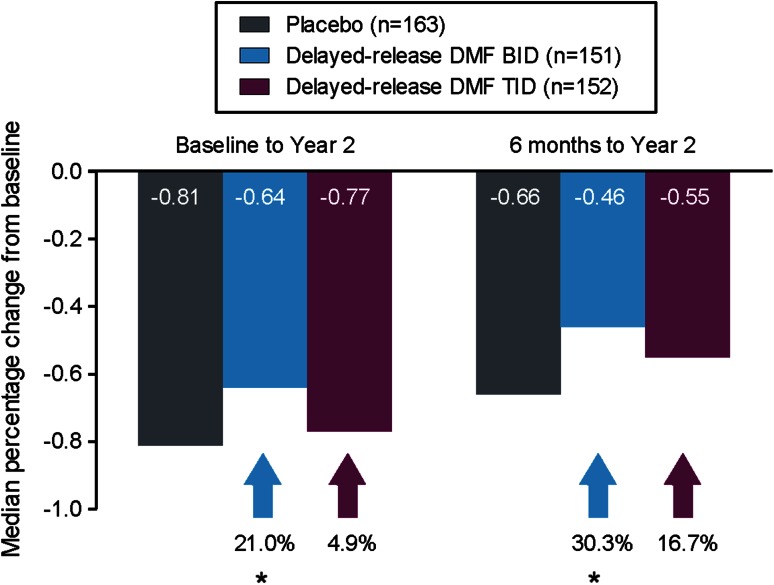
Median percentage change in whole brain volume at 2 years relative to baseline and 6 months. Comparisons vs. placebo were based on analysis of covariance on ranked data, adjusted for region and normalized brain volume at 6 months. *P < 0.05 vs. placebo. *BID* twice daily, *DMF* dimethyl fumarate, *TID* three times daily

The median PBVC in the placebo group was −0.81 % from baseline to 2 years and −0.66 % from 6 months to 2 years (Fig. 4; Table e-5). Delayed-release DMF BID and TID attenuated brain volume loss from baseline to 2 years by 21 % (*P* = 0.0449) and 5 % (*P* = 0.6398), respectively, compared with placebo. Similar results were observed from 6 months to 2 years with relative reductions of 30 % (*P* = 0.0214) and 17 % (*P* = 0.2478) with delayed-release DMF BID and TID, respectively.

## Discussion

Among the results of the DEFINE study were the findings that delayed-release DMF significantly reduced the number of new or enlarging T2-hyperintense lesions and Gd+ lesions at 2 years compared with placebo, suggesting reductions in both the inflammatory processes that correlate with MS relapses and the burden of focal white matter disease [12]. The analysis presented here expands on these results by assessing the effects of delayed-release DMF on additional MRI measures, providing information on the time course of these effects, and evaluating the generality of the effects across a diverse patient population. Reductions in the number of new or enlarging T2-hyperintense lesions and Gd+ lesions in patients treated with delayed-release DMF compared with placebo were observed as early as the first scheduled MRI assessment at 6 months and were sustained at 1 and 2 years; reductions were also seen in the number of T1-hypointense lesions at 6 months, 1 and 2 years. Compared with placebo, delayed-release DMF reduced the volume of T2-hyperintense and Gd+ lesions beginning at 6 months and the volume of T1-hypointense lesions beginning at 1–2 years. In support of these findings, analyses of brain atrophy revealed a significant reduction in PBVC from 6 months to 2 years in patients treated with delayed-release DMF BID compared with placebo. The effects on delayed-release DMF on the number of new or enlarging T2-hyperintense lesions and Gd+ lesion activity were seen across patient subpopulations representing a broad range of demographic and disease characteristics. The validity of these findings is further supported by sensitivity analyses using observed data without imputation. Altogether, the reductions in MRI disease activity with delayed-release DMF treatment are suggestive of an early decrease in brain inflammation and reduction of tissue destruction and axonal loss associated with the progression of disability.

The observations in DEFINE are consistent with those seen in a Phase 2 dose-ranging study, in which delayed-release DMF 240 mg TID significantly reduced the mean total number of new Gd+ lesions by 69 % from 3 to 6 months, compared with placebo [3]. The results of the present study are also consistent with those reported in CONFIRM, in which delayed-release DMF BID and TID treatment reduced the mean number of new or enlarging T2-hyperintense lesions by 71 and 73 %, Gd+ lesion activity by 74 and 65 %, and the mean number of new T1-hypointense lesions by 57 and 65 %, respectively (all *P* < 0.001) [13]. The reductions in lesion number with delayed-release DMF relative to placebo were generally greater than those seen with glatiramer acetate, used as a reference comparator in CONFIRM [13]. The effects of delayed-release DMF on MRI lesion outcomes in the present study are comparable with, or in some cases exceed, those of other oral [6, 7, 18, 19] and parenteral [20, 21] disease-modifying therapies in 2-year, Phase 3 RRMS studies, although cross-study comparisons should be interpreted with caution due to differences in study design, population, and MRI methodology.

Examination of a range of study subpopulations defined by baseline disease characteristics or demographics showed that delayed-release DMF treatment conferred MRI benefits across patient groups with RRMS, a result that expands on the observed decrease in new Gd+ lesion development seen across subgroups in the Phase 2 study [18] and is consistent with findings in the CONFIRM study [13].

Measurements of brain atrophy continue to be an area with evolving methodologies. Reported slowing of rates of atrophy with disease-modifying therapies may vary depending on the therapy [22], whether the rate of atrophy is expressed using whole brain volume as the denominator (FREEDOMS [23] and ALLEGRO studies [20]) or brain parenchymal fraction (AFFIRM [24] and TEMSO [25]), and with the method used to measure the atrophy [26]. Even when utilizing the same overall methodology, the area of the brain included in the calculation can differ, for example, if the MRI scans do not provide full brain coverage [21]. Thus, comparing across trials for this endpoint is especially problematic. Despite a low rate of brain atrophy in the placebo group in DEFINE, a beneficial and statistically significant effect on atrophy was seen with the BID dose regimen at 2 years when evaluated either from baseline or from 6 months. Although not observed in this study, some therapies for MS have shown greater decreases in brain volume compared with placebo in the first months following initiation of treatment, a phenomenon sometimes referred to as ‘pseudoatrophy’. However, the mechanisms responsible for this are unclear, and despite considering this effect to be related to decreasing brain inflammation, it is not always seen in clinical trials of agents with potent anti-inflammatory effects [21].

## Conclusion

Overall, expanded results of MRI analyses from the DEFINE study demonstrate rapid efficacy of delayed-release DMF in terms of reduced lesion number and volume compared with placebo, consistent with evidence from a Phase 2 study [3] and the Phase 3 CONFIRM study [13]. Reductions in lesion counts and volume, together with supportive data on brain atrophy, are consistent with the hypothesis that delayed-release DMF favorably affects multiple aspects of MS pathophysiology.

## Electronic supplementary material

Below is the link to the electronic supplementary material.

Supplementary material 1 (DOCX 17292 kb)

Supplementary material 2 (TIFF 78 kb)

Supplementary material 3 (TIFF 8570 kb)

Supplementary material 4 (TIFF 8591 kb)
