# Supplementary material for: Effects of delayed-release dimethyl fumarate on MRI measures in the Phase 3 DEFINE study
Source: J Neurol. 2014 Jul 3;261(9):1794–802. doi: 10.1007/s00415-014-7412-x (PMC4155185; doi:10.1007/s00415-014-7412-x)
Supplement: Supplementary file 1 — Supplementary material 1 (DOCX 17292 kb) [file 415_2014_7412_MOESM1_ESM.docx]

# ONLINE SUPPLEMENT

# APPENDIX e-1: STUDY PERSONNEL

## Advisory Committee

Ralf Gold (Coordinating Investigator and Chairperson), Ludwig Kappos, Douglas Arnold, Amit Bar-Or, Gavin Giovannoni, Krzystof Selmaj (Study Investigators), Katherine T. Dawson (Senior Medical Director, Biogen Idec), Phillip Gallacher (Clinical Program Lead, Biogen Idec), Carole Milla, (Clinical Trial Manager, Biogen Idec), Minhua Yang (Project Statistician, Biogen Idec).

## Data Safety Monitoring Committee

Jack Antel (Chairperson, Neurology; Montreal Neurological Hospital, Montreal, Quebec, Canada), George Bakris (Nephrology; University of Chicago, Pritzker School of Medicine, Chicago, IL, USA), Raymond Chung (Hepatology; Massachusetts General Hospital, Boston, MA, USA), Peter R. Kowey (Cardiology; Jefferson Medical College, Wynnewood, Pennsylvania, USA), Chris Polman (Neurology; VU Medical Center, Amsterdam, The Netherlands), John Richert (thru February 2010: Neurology; National Multiple Sclerosis Society, New York, NY, USA), Burt Seibert (Independent Biostatistician; Statistical Services Network, Plaistow, NH, USA), James Ware (Statistics; Harvard School of Public Health, Boston, MA, USA).

## Independent Neurology Evaluation Committee

David Brandes (Northridge Multiple Sclerosis Center, Northridge, CA, USA), David Brassat (INSERM U.563, Hôpital Purpan, Toulouse, France), Bruce Cohen (Fineberg School of Medicine, Chicago, IL, USA), Ricarda Diem (University of the Saarland, Homburg/Saar, Germany), Myla Goldman (University of Virginia, Charlottesville, VA, USA), Robert Herndon (University of Mississippi Medical Center, Jackson, MS, USA), Aaron Miller (Mount Sinai Medical Center, New York, NY, USA), Hayrettin Tumani (University of Ulm, Ulm, Germany).

## Principal Investigators

**Australia:** Michael Barnett, Helmut Butzkueven, Roy Beran, Reynolds Casse, Caron Chapman, Jeannette Lechner-Scott, Richard Macdonell, Mark Paine, Raymond Schwartz; **Austria:** Thomas Berger, Franz Fazekas, Gerhard Ransmayr, Karl Vass; **Belgium:** Shibeshih Belachew, Danny Decoo, Peter Paul De Deyn, Bénédicte Dubois, Luc Vande Gaer, Robert Medaer, Pierrette Seeldrayers, Christian Sindic, Ludo Vanopdenbosch; **Bosnia Herzegovina:** Sanja Grgic; **Canada:** Virender Bhan, Jean-Pierre Bouchart, Suzanne Christie, Amit Bar-Or, François Jacques, Pierre Grammond, Felix Veloso, Galina Vorobeychick; **Croatia:** Vesna Brinar, Vida Demarin, Josip Rudež, Silva Soldo-Butković, Ranka Baraba Vurdelja; **Czech Republic:** Zdeněk Ambler, David Doležil, Eva Havrdova, Petr Kanovsky, Eva Meluzinova, Jiří Nova'k, Ivan Rektor, Ondrej Skoda, Marta Vachová; **France:** William Camu, Pierre Clavelou, Antoanela Irène Coman, Christian Confavreux, Gilles Edan, Olivier Gout, Christine Lebrun-Frenay, Anne-Caroline Papeix, Patrick Vermersch; **Germany:** Katrin Bachus-Banaschak, Martin Berghoff, Florian Bethke, Hans-Jürgen Boldt, Andrew Chan, Hans-Christoph Diener, Ilonka Eisensehr, Peter Emrich, Bernd Griewing, Judith Haas, Christoph Heesen, Fedor Heidenreich, Frank Hoffmann, Andreas Hufnagel, Ingo Kleiter, Wilfried Lüer, Silke Marckmann-Böenke, Martin Marziniak, Patrick Oschmann, Thorsten Rosenkranz, Hauke Schneider, Veneta Siefjediers, Joachim Springub, Martin Stangel, Florian Stögbauer, Florian Then Bergh, Klaus Tiel-Wilck, Konstanze Tinschert; **Greece:** Nikolaos Grigoriadis, Clementine Karageorgiou, Athanssios Kyritsis, Alexandros Papadimitriou, Thomas Thomaides, Nicholas Vlaikidis; **Guatemala:** David Yaxcal Chon, Luis Fernando Salguero Gonzalez, Hugo Alfredo Ordoñez Sarg; **India:** CS Agrawal, Madhuri Behari, Velmurugendran Cu , Shamsher Dwivedee, Sudhir Kothari, Rahul Kulkarni, Suman Kushwaha, AK Meena, MM Mehndiratta, Subhash Chandra Mukherjee, Lekha Pandit, Arun Shah, Rakesh Shukla, K Vijayan; **Israel:** Anat Achiron, Shlomo Flechter, Ron Milo, Adi Vaknin-Dembinsky; **Italy:** Carlo Pozzilli; **Mexico:** José Luis Oropeza de Alba, Freddy Guillermo Castro Farfan, Ricardo Alberto Rangel Guerra, Ildefonso Rodriguez Leyva, Raúl Revilla, Jose Flores Rivera; **Moldova:** Mihail Gavriliuc, Stanislav Groppa, Olesea Odainic; **Netherlands:** Raymond Hupperts, EACM Sanders; **New Zealand:** Deborah Mason, Paul Timmings, Ernest Willoughby; **Poland:** Anna Czlonkowska, Malgorzata Dorobek, Wieslaw Drozdowski, Hanka Hertmanowska, Waldemar Fryze, Magdalena Kleczkowska, Jan Kochanowicz, Hubert Kwieciński, Przemyslaw Nowacki, Ryszard Podemski, Andrzej Potemkowski, Krzysztof Selmaj, Zbigniew Stelmasiak, Andrzej Wajgt, Tomasz Zielinski; **Republic of Macedonia:** Vera Daskalovska; **Romania:** Alexandru Ovidiu Bajenaru, Fior Dafin Muresanu, Sanda Maria Nica, Mihaela Adriana Simu; **Serbia:** Svetlana Miletić Drakulić, Congor Nadj, Dragana Obradovic, Slobodan Vojinovic; **Slovakia:** Edita Kahancová, Viliam Krajnak, Egon Kurca, L'ubomir Lisý, Peter Turčáni; **South Africa:** Frans Badenhorst, Judy Green, Jeannine Heckmann, Michael Isaacs; **Switzerland:** Ludwig Kappos, Michael Linnebank, Stefanie Müller; **Ukraine:** Nataliya Buchakchyys'ka, Alla Goloborodko, Tetyana Kobys, Volodymyr Lebedynets, Nataliya Lytvynenko, Tetyana Nehrych, Igor Pasyura, Tatyana Ryabichenko, Nataliya Voloshina; **United Kingdom:** Martin Duddy, Clive Hawkins, Richard Nicholas, Jacqueline Palace, Basil Sharrack, Eli Silber, Ben Turner; **United States:** Ann Camac, Warren Chumley, Heidi Crayton, Shanker Dixit, Jeffrey English, Keith Edwards, P. Steven Freedman, Suzanne Gazda, Lawrence Goldstick, Norman Gordon, Christopher Gottschalk, Erica Grazioli, Bart Grelinger, Barbara Green, Ghazala Hayat, Barry Hendin, Todd Janus, LilY Jung Henson, Lawrence Goldstick, William Grainger, Mark Gudesblatt, Ajay Gupta, Afif Hentati, Craig Herrman, Arthur Itkin, Lloyd Kasper, Ellen Lathi, Ellen Mowry, Gareth Parry, Allan Perel, T. Hemanth Rao, Peter Riskind, Syed Rizvi, Michael Rossen, Howard Rossman, Thomas Scott, S. James Shafer, James Storey, Jr., Ben Thrower, Carlo Tornatore, William Tosches, Lori Trefts, Jr., Anthony Turel, Jr., Bianca Weinstock-Guttman.

# APPENDIX e-2: SUPPLEMENTARY TABLES AND FIGURES

## Table e-1. Key baseline characteristics of the MRI cohort and non-MRI cohort.

| Characteristic | MRI cohort  (n=540) | Non-MRI cohort  (n=697) |
| --- | --- | --- |
| Age, years |  |  |
| Mean | 38.4 | 38.5 |
| Median | 39.0 | 39.0 |
| Female, n (%) | 417 (77) | 494 (71) |
| Prior approved MS treatment, n (%)^a^ | 219 (41) | 283 (41) |
| Time since first MS symptoms, years |  |  |
| Mean | 8.4 | 8.2 |
| Median | 7.0 | 7.0 |
| Relapses in previous year |  |  |
| Mean | 1.3 | 1.3 |
| Median | 1.0 | 1.0 |
| EDSS score^b^ |  |  |
| Mean | 2.4 | 2.5 |
| Median | 2.0 | 2.5 |

Abbreviations: EDSS = Expanded Disability Status Scale; MS = multiple sclerosis.

^a^Interferon beta-1a, interferon beta-1b, glatiramer acetate, and natalizumab. Patients may have received more than one prior MS medication. Patients may also have received other non-approved therapies for MS.

^b^Score on the EDSS scale range from 0 to 10, with higher scores indicating greater degree of disability.

## Table e-2. MRI outcomes during the 96-week study (intent-to-treat MRI cohort): new or enlarging T2 lesions.

| MRI assessment | Placebo  (n=165) | Delayed-release DMF BID (n=152) | Delayed-release DMF TID (n=152) |
| --- | --- | --- | --- |
| **T2 lesion numbers** |  |  |  |
| Number of new or enlarging T2 lesions from baseline to 6 months (week 24)^a^ | | | |
| Mean (SD) | 5.3 (9.01) | 1.3 (3.06) | 2.0 (5.21) |
| Median (25^th^, 75^th^ percentile) | 2.0 (0.0, 6.0) | 0.0 (0.0, 1.0) | 0.5 (0.0, 2.0) |
| Adjusted mean | 5.2 | 1.1 | 1.6 |
| Lesion mean ratio (95% CI) |  | 0.20 (0.14, 0.30) | 0.31 (0.21, 0.45) |
| % change vs placebo (95% CI) |  | -80 (-86, -70) | -69 (-79, -55) |
| *p*-value |  | <0.0001 | <0.0001 |
| Number of new or enlarging T2 lesions from baseline to 1 year (week 48)^a^ | | | |
| Mean (SD) | 10.1 (18.32) | 2.0 (4.78) | 3.0 (7.43) |
| Median (25^th^, 75^th^ percentile) | 4.0 (0.0, 12.0) | 0.0 (0.0, 2.0) | 1.0 (0.0, 2.5) |
| Adjusted mean | 10.3 | 1.6 | 2.6 |
| Lesion mean ratio (95% CI) |  | 0.16 (0.11, 0.24) | 0.25 (0.17, 0.37) |
| % change vs placebo (95% CI) |  | -84 (-89, -76) | -75 (-83, -63) |
| *p*-value |  | <0.0001 | <0.0001 |
| Number of new or enlarging T2 lesions from baseline to 2 years (week 96)^a^ | | | |
| Mean (SD) | 16.5 (23.40) | 3.2 (7.61) | 4.9 (11.50) |
| Median (25^th^, 75^th^ percentile) | 7.0 (0.0, 20.0) | 1.0 (0.0, 3.0) | 1.0 (0.0, 5.0) |
| Adjusted mean | 17.0 | 2.6 | 4.4 |
| Lesion mean ratio (95% CI) |  | 0.15 (0.10, 0.23) | 0.26 (0.17, 0.38) |
| % change vs placebo (95% CI) |  | -85 (-90, -77) | -74 (-83, -62) |
| *p*-value |  | <0.0001 | <0.0001 |
| Patients with new or enlarging T2 lesions at 2 years (week 96), n (%) | | | |
| 0 lesions | 45 (27) | 68 (45) | 62 (41) |
| 1 lesion | 8 (5) | 26 (17) | 28 (18) |
| 2 lesions | 3 (2) | 14 (9) | 11 (7) |
| 3 lesions | 8 (5) | 10 (7) | 5 (3) |
| ≥4 lesions | 101 (61) | 34 (22) | 46 (30) |
| **T2 lesion volumes** |  |  |  |
| Volume of T2 lesions at 6 months (week 24), mm^3^ | | | |
| Mean (SD) | 6788.7 (7952.80) | 8302.9 (9498.36) | 9098.9 (11280.51) |
| Median (min, max) | 4266.0 (6, 51061) | 4991.5 (9, 50746) | 5218.5 (17, 93131) |
| Change from baseline to 6 months (week 24) | | | |
| Mean (SD) | 305.4 (2773.09) | –245.3 (1376.00) | –349.4 (2311.19) |
| Median (min, max) | 37.0 (–3951, 33505) | –77.5 (–6171, 6704) | –39.5 (–24402, 4853) |
| *p*-value^b^ |  | 0.0016 | 0.0108 |
| % change from baseline to 6 months (week 24)^c^ | | | |
| Mean (SD) | 15.9 (129.10) | -2.3 (12.85) | 2.9 (53.20) |
| Median (25^th^, 75^th^ percentile) | 1.6 (-5.4, 11.3) | -3.5 (-9.0, 3.1) | -1.7 (-8.1, 4.0) |
| *p*-value^b^ |  | 0.0002 | 0.0035 |
| Volume of T2 lesions at 1 year (week 48), mm^3^ | | | |
| Mean (SD) | 6756.8 (7269.82) | 7703.2 (8372.51) | 8895.0 (10443.03) |
| Median (min, max) | 5347.0 (0, 51450) | 5506.0 (9, 46959) | 6134.0 (14, 89523) |
| Change from baseline to 1 year (week 48) | | | |
| Mean (SD) | 261.4 (1493.54) | –430.5 (1397.78) | –496.2 (2495.35) |
| Median (min, max) | 151.0 (–5854, 10422) | –254.5 (–5968, 6961) | –193.0 (–24248, 9849) |
| *p*-value^b^ |  | <0.0001 | <0.0001 |
| % change from baseline to 1 year (week 48)^c^ | | | |
| Mean (SD) | 11.7 (48.77) | -4.7 (15.21) | 0.1 (35.01) |
| Median (25^th^, 75^th^ percentile) | 6.5 (-5.5, 14.9) | -5.8 (-12.3, 1.9) | -3.7 (-9.3, 3.5) |
| *p*-value^b^ |  | <0.0001 | <0.0001 |
| Volume of T2 lesions at 2 years (week 96), mm^3^ | | | |
| Mean (SD) | 6898.1 (6684.14) | 7631.9 (8100.39) | 8784.6 (10062.30) |
| Median (min, max) | 6898.1 (29, 47519) | 6337.5 (6, 48154) | 6952.5 (14, 85685) |
| Change from baseline to 2 years (week 96) | | | |
| Mean (SD) | 386.0 (1675.68) | –365.1 (1428.74) | –361.6 (1584.39) |
| Median (min, max) | 386.0 (–8978, 10330) | –323.0 (–7230, 9867) | –289.0 (–13165, 6283) |
| *p*-value^b^ |  | <0.0001 | <0.0001 |
| % change from baseline to 2 years (week 96)^c^ | | | |
| Mean (SD) | 20.4 (67.28) | -6.2 (16.84) | 0.8 (32.65) |
| Median (25^th^, 75^th^ percentile) | 20.1 (-3.4, 20.4) | -6.2 (-12.9, 1.5) | -1.9 (-10.8, 1.4) |
| *p*-value^b^ |  | <0.0001 | <0.0001 |

Abbreviations: BID = twice daily; DMF = dimethyl fumarate; TID = three times daily.

^a^Percentage change, 95% CI and *p*-value for comparison between active and placebo groups, based on negative binomial regression, adjusted for region and baseline volume of T2 lesions.

^b^*p*-value for comparison between active and placebo groups, based on analysis of covariance on ranked data, adjusted for region and baseline T2 lesion volume.

^c^Percentage change calculated for patients with non-zero volume data in the denominator.

## Table e-3. MRI outcomes during the 96-week study (intent-to-treat MRI cohort): Gd+ lesions.

| MRI assessment | Placebo  (n=165) | Delayed-release DMF BID (n=152) | Delayed-release DMF TID (n=152) |
| --- | --- | --- | --- |
| **Gd+ lesion number** |  |  |  |
| Number of Gd+ lesions at 6 months (week 24)^a^ | | | |
| Mean (unadjusted 95% CI)^b^ | 1.5 (1.0, 2.0) | 0.1 (0.0, 0.1) | 0.3 (0.0, 0.6) |
| Median (25^th^, 75^th^ percentile) | 0.0 (0.0, 2.0) | 0.0 (0.0, 0.0) | 0.0 (0.0, 0.0) |
| Odds ratio (95% CI) |  | 0.06 (0.02, 0.13) | 0.19 (0.11, 0.35) |
| *p*-value |  | <0.0001 | <0.0001 |
| Number of Gd+ lesions at 1 year (week 48)^a^ | | | |
| Mean (unadjusted 95% CI)^b^ | 1.4 (1.0, 1.9) | 0.1 (0.0, 0.2) | 0.4 (0.0, 0.9) |
| Median (25^th^, 75^th^ percentile) | 0.0 (0.0, 2.0) | 0.0 (0.0, 0.0) | 0.0 (0.0, 0.0) |
| Odds ratio (95% CI) |  | 0.08 (0.04, 0.17) | 0.13 (0.07, 0.25) |
| *p*-value |  | <0.0001 | <0.0001 |
| Number of Gd+ lesions at 2 years (week 96)^a^ | | | |
| Mean (unadjusted 95% CI)^b^ | 1.8 (1.1, 2.4) | 0.1 (0.0, 0.2) | 0.5 (0.2, 0.8) |
| Median (25^th^, 75^th^ percentile) | 0.0 (0.0, 2.0) | 0.0 (0.0, 0.0) | 0.0 (0.0, 0.0) |
| Odds ratio (95% CI) |  | 0.10 (0.05-0.22) | 0.27 (0.15-0.46) |
| *p*-value |  | <0.0001 | <0.0001 |
| Patients with Gd+ lesions at 2 years (week 96), n (%) | | | |
| 0 lesions | 103 (62) | 142 (93) | 130 (86) |
| 1 lesion | 16 (10) | 8 (5) | 10 (7) |
| 2 lesions | 13 (8) | 1 (<1) | 2 (1) |
| 3–4 lesions | 15 (9) | 0 | 3 (2) |
| ≥5 lesions | 18 (11) | 1 (<1) | 7 (5) |
| **Gd+ lesion volume** |  |  |  |
| Volume of Gd+ lesions at 6 months (week 24), mm^3^ | | | |
| Mean (SD) | 147.4 (393.56) | 6.2 (36.95) | 38.7 (219.03) |
| Median (min, max) | 0.0 (0, 3204) | 0.0 (0, 381) | 0.0 (0, 2443) |
| Change from baseline 6 months (week 24) | | | |
| Mean (SD) | –1.8 (396.23) | –203.2 (672.69) | –118.7 (512.08) |
| Median (25^th^, 75^th^ percentile) | 0.0 (–34.0, 0.0) | 0.0 (–41.5, 0.0) | 0.0 (–46.0, 0.0) |
| *p*-value^c^ |  | 0.0059 | 0.0176 |
| % change from baseline to 6 months (week 24)^d^ | | | |
| Mean (SD) | 14.1 (200.82) | -95.0 (22.71) | -82.0 (45.20) |
| Median (min, max) | -56.4 (-100, 955) | -100.0 (-100, 49) | -100.0 (-100, 108) |
| *p*-value^c^ |  | <0.0001 | <0.0001 |
| Volume of Gd+ lesions at 1 year (week 48), mm^3^ | | | |
| Mean (SD) | 129.0 (259.97) | 15.3 (75.52) | 36.2 (245.78) |
| Median (min, max) | 0.0 (0, 1459) | 0.0 (0, 555) | 0.0 (0, 2781) |
| Change from baseline to 1 year (week 48) | | | |
| Mean (SD) | –12.6 (315.15) | –160.9 (576.88) | –110.2 (539.57) |
| Median (25^th^, 75^th^ percentile) | 0.0 (–12.6, 0.0) | 0.0 (–78.5, 0.0) | 0.0 (–84.0, 0.0) |
| *p*-value^c^ |  | 0.0063 | 0.0019 |
| % change from baseline to 1 year (week 48)^d^ | | | |
| Mean (SD) | 97.4 (677.50) | –88.5 (42.78) | –65.9 (140.48) |
| Median (min, max) | –56.4 (–100, 5453) | –100.0 (–100, 128) | –100.0 (–100, 809) |
| *p*-value^c^ |  | <0.0001 | <0.0001 |
| Volume of Gd+ lesions at 2 years (week 96), mm^3^ | | | |
| Mean (SD) | 145.6 (365.54) | 20.4 (167.71) | 56.5 (223.42) |
| Median (min, max) | 63.0 (0, 3736) | 0.0 (0, 2034) | 0.0 (0, 2223) |
| Change from baseline to 2 years (week 96) | | | |
| Mean (SD) | 15.1 (404.04) | –152.7 (576.36) | –57.8 (336.28) |
| Median (25^th^, 75^th^ percentile) | 0.0 (0.0, 15.1) | 0.0 (–152.7, 0.0) | 0.0 (–57.8, 0.0) |
| *p*-value^c^ |  | <0.0001 | <0.0001 |
| % change from baseline to 2 years (week 96)^d^ | | | |
| Mean (SD) | 106.4 (389.53) | –79.0 (113.62) | –52.6 (114.40) |
| Median (min, max) | –31.3 (–100, 2192) | –100.0 (–100, 698) | –100 (–100, 481) |
| *p*-value^c^ |  | <0.0001 | <0.0001 |

Abbreviations: BID = twice daily; DMF = dimethyl fumarate; Gd+ = gadolinium-enhancing; TID = three times daily.

^a^Odds ratio and *p*-value for comparison between active and placebo groups based on ordinal logistic regression, adjusted for region and baseline number of Gd+ lesions.

^b^95% CI of the mean is calculated using t distribution under the large sample assumption.

^c^*p*-value for comparison between active and placebo groups, based on analysis of covariance on ranked data, adjusted for region and baseline Gd+ lesion volume.

**^d^**Percentage change calculated for patients with non-zero volume data in the denominator.

## Table e-4. MRI outcomes during the 96-week study (intent-to-treat MRI cohort): T1-hypointense (non-enhancing) lesions.

| MRI assessment | Placebo  (n=165) | Delayed-release DMF BID (n=151) | Delayed-release DMF TID (n=152) |
| --- | --- | --- | --- |
| **T1-hypointense lesion number** |  |  |  |
| Number of new T1-hypointense lesions from baseline to 6 months (week 24)^a^ | | | |
| Mean (SD) | 2.0 (3.52) | 1.0 (2.30) | 1.2 (2.56) |
| Median (25^th^, 75^th^ percentile) | 0.0 (0.0, 3.0) | 0.0 (0.0, 1.0) | 0.0 (0.0, 1.0) |
| Adjusted mean | 1.9 | 0.8 | 1.0 |
| Lesion mean ratio (95% CI) |  | 0.42 (0.29, 0.61) | 0.52 (0.36, 0.75) |
| % change vs placebo (95% CI) |  | -58 (-71, -39) | -48 (-64, -25) |
| *p*-value |  | <0.0001 | 0.0005 |
| Number of new T1-hypointense lesions from baseline to 1 year (week 48)^a^ | | | |
| Mean (SD) | 3.5 (5.59) | 1.4 (2.88) | 1.7 (3.20) |
| Median (25^th^, 75^th^ percentile) | 1.0 (0.0, 4.0) | 1.0 (0.0, 2.0) | 1.0 (0.0, 2.0) |
| Adjusted mean | 3.5 | 1.1 | 1.4 |
| Lesion mean ratio (95% CI) |  | 0.31 (0.22, 0.44) | 0.39 (0.28, 0.56) |
| % change vs placebo (95% CI) |  | -69 (-78, -56) | -61 (-72, -44) |
| *p*-value |  | <0.0001 | <0.0001 |
| Number of new T1-hypointense lesions from baseline to 2 years (week 96)^a^ | | | |
| Mean (SD) | 5.7 (8.32) | 2.0 (4.13) | 2.5 (4.82) |
| Median (25^th^, 75^th^ percentile) | 2.0 (0.0, 8.0) | 1.0 (0.0, 3.0) | 1.0 (0.0, 3.0) |
| Adjusted mean | 5.6 | 1.5 | 2.1 |
| Lesion mean ratio (95% CI) |  | 0.28 (0.20, 0.39) | 0.37 (0.26, 0.52) |
| % change vs placebo (95% CI) |  | -72 (-80, -61) | -63 (-74, -48) |
| *p*-value |  | <0.0001 | <0.0001 |
| Patients with new T1-hypointense lesions at 2 years (week 96), n (%) | | | |
| 0 lesions | 59 (36) | 61 (40) | 69 (45) |
| 1 lesion | 16 (10) | 35 (23) | 29 (19) |
| 2 lesions | 10 (6) | 15 (10) | 13 (9) |
| 3–4 lesions | 19 (12) | 26 (17) | 18 (12) |
| ≥5 lesions | 61 (37) | 14 (9) | 23 (15) |
| **T1-hypointense lesion volume** |  |  |  |
| Volume of T1-hypointense lesions at 6 months (week 24), mm^3^ | | | |
| Mean (SD ) | 2351.4 (4056.49) | 3303.6 (5370.93) | 3485.8 (5052.24) |
| Median (min, max) | 1084.0 (0, 38267) | 1293.0 (0, 37602) | 1460.5 (0, 34144) |
| Change from baseline to 6 months (week 24) | | | |
| Mean (SD) | 53.1 (779.89) | 140.1 (1401.04) | 17.1 (1043.78) |
| Median (min, max) | 14.0 (–4092, 5502) | 16.0 (–4709, 14995) | 16.0 (–8062, 3845) |
| *p*-value^b^ |  | 0.8232 | 0.5332 |
| % change from baseline to 6 months (week 24)^c^ | | | |
| Mean (SD) | 21.5 (170.09) | 6.4 (30.24) | 18.8 (109.37) |
| Median (25^th^, 75^th^ percentile) | 4.3 (-12.5, 21.8) | 1.5 (-9.7, 16.7) | 2.5 (-11.8, 18.5) |
| *p*-value^b^ |  | 0.7559 | 0.9356 |
| Volume of T1-hypointense lesions at 1 year (week 48), mm^3^ | | | |
| Mean (SD ) | 2486.8 (4073.82) | 3155.9 (5164.18) | 3447.4 (4556.22) |
| Median (min, max) | 1436.0 (0, 39551) | 1351.5 (0, 39671) | 1980.0 (0, 28856) |
| Change from baseline to 1 year (week 48) | | | |
| Mean (SD) | 146.9 (691.21) | 152.2 (1558.64) | 37.5 (1017.57) |
| Median (min, max) | 69.0 (–4927, 2909) | 26.0 (–4715, 17064) | 37.5 (–5375, 4992) |
| *p*-value^b^ |  | 0.1523 | 0.1155 |
| % change from baseline to 1 year (week 48)^c^ | | | |
| Mean (SD) | 20.6 (54.44) | 5.8 (28.54) | 14.5 (53.35) |
| Median (25^th^, 75^th^ percentile) | 11.6 (-7.1, 30.2) | 5.4 (-9.2, 18.1) | 4.7 (-10.6, 19.5) |
| *p*-value^b^ |  | 0.0126 | 0.0783 |
| Volume of T1-hypointense lesions at 2 years (week 96), mm^3^ | | | |
| Mean (SD) | 2731.9 (3922.19) | 3257.6 (5197.03) | 3593.6 (4847.95) |
| Median (min, max) | 2731.9 (0, 37425) | 1705.5 (0, 43133) | 2068.5 (0, 34084) |
| Change from baseline to 2 years (week 96) | | | |
| Mean (SD) | 279.3 (944.18) | 218.8 (1801.45) | 268.2 (912.55) |
| Median (min, max) | 279.3 (–6902, 4955) | 86.0 (–4704, 20526) | 139.5 (–2735, 4460) |
| *p*-value^b^ |  | 0.0003 | 0.0286 |
| % change from baseline to 2 years (week 96)^c^ | | | |
| Mean (SD) | 26.9 (49.05) | 8.4 (38.85) | 21.8 (59.96) |
| Median (25^th^, 75^th^ percentile) | 26.9 (-3.4, 27.5) | 8.4 (-7.2, 17.0) | 12.7 (-5.0, 21.8) |
| *p*-value^b^ |  | <0.0001 | 0.0063 |

Abbreviations: BID = twice daily; DMF = dimethyl fumarate; TID = three times daily.

^a^Percentage change, 95% CI and *p*-value for comparison between the active and placebo groups based on negative binomial regression, adjusted for region and baseline volume of T1-hypointense lesions.

^b^*p*-value for comparison between active and placebo groups, based on analysis of covariance on ranked data, adjusted for region and baseline T1-hypointense lesion volume.

^c^Percentage change calculated for patients with non-zero volume data in the denominator.

## Table e-5. Whole brain volume outcomes during the 96-week study (intent-to-treat MRI cohort).

| Assessment | Placebo  (n=163) | Delayed-release DMF BID  (n=151) | Delayed-release DMF TID (n = 152) |
| --- | --- | --- | --- |
| % change from 6 months (week 24) to 1 year (week 48) | | | |
| Mean (SD) | -0.248 (0.5243) | -0.229 (0.5052) | -0.256 (0.5067) |
| Median (min, max) | -0.260 (-2.10, 1.07) | -0.220 (-1.74, 1.93) | -0.240 (-1.71, 1.07) |
| % reduction vs placebo^a^ |  | 15.4 | 7.7 |
| *p*-value^b^ |  | 0.6065 | 0.8982 |
| % change from 6 months (week 24) to 2 years (week 96) | | | |
| Mean (SD) | -0.775 (1.0115) | -0.598 (0.9594) | -0.716 (0.8978) |
| Median (min, max) | -0.660 (-4.62, 2.22) | -0.460 (-4.35, 3.91) | -0.550 (-4.32, 1.65) |
| % reduction vs placebo^a^ |  | 30.3 | 16.7 |
| *p*-value^b^ |  | 0.0214 | 0.2478 |
| % change from baseline to 6 months (week 24) | | | |
| Mean (SD) | -0.177 (0.5265) | -0.202 (0.5840) | -0.251 (0.5523) |
| Median (min, max) | -0.180 (-1.54, 1.53) | -0.180 (-1.77, 1.36) | -0.200 (-1.69, 1.03) |
| % reduction vs placebo^a^ |  | 0.0 | -11.1 |
| *p*-value^b^ |  | 0.9282 | 0.5082 |
| % change from baseline to 1 year (week 48) | | | |
| Mean (SD) | -0.425 (0.7724) | -0.430 (0.7635) | -0.507 (0.7659) |
| Median (min, max) | -0.380 (-3.08, 2.14) | -0.360 (-2.90, 2.20) | -0.405 (-2.88, 1.10) |
| % reduction vs placebo^a^ |  | 5.3 | -6.6 |
| *p*-value^c^ |  | 0.7699 | 0.7870 |
| % change from baseline to 2 years (week 96) | | | |
| Mean (SD) | -0.952 (1.2971) | -0.800 (1.1987) | -0.968 (1.1595) |
| Median (min, max) | -0.810 (-6.16, 2.96) | -0.640 (-5.80, 3.96) | -0.770 (-5.76, 2.20) |
| % reduction vs placebo^a^ |  | 21.0 | 4.9 |
| *p*-value^c^ |  | 0.0449 | 0.6398 |

Abbreviations: BID = twice daily; DMF = dimethyl fumarate; TID = three times daily.

^a^Based on median.

^b^*p*-value for comparison between active and placebo groups, based on analysis of covariance on ranked data, adjusted for region and brain volume at week 24.

^c^*p*-value for comparison between active and placebo groups, based on analysis of covariance on ranked data, adjusted for region and baseline brain volume.

## Figure e-1

MRI lesion number outcomes – results of primary and sensitivity analyses

Sensitivity analyses, illustrating outcomes based on all observed data alongside those based on observed data prior to starting alternative MS medication in patients who switched MS therapy.


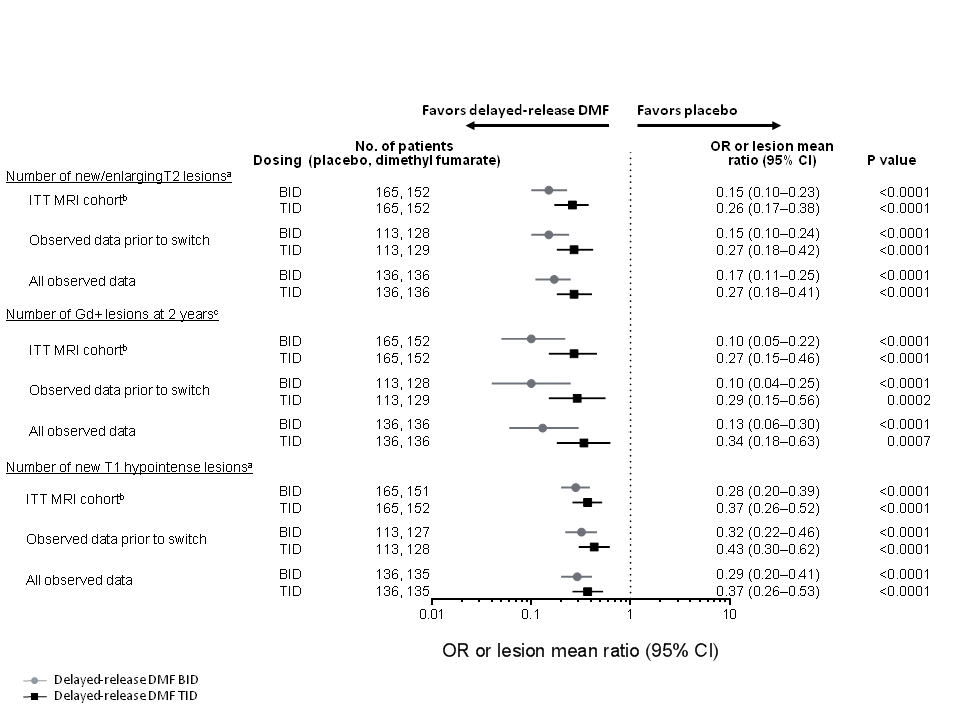


^a^Ratios shown are lesion mean ratios, from baseline to 2 years; 95% CI and *p*-value for comparison between active and placebo groups, based on negative binomial regression, adjusted for region and baseline lesion volume.

^b^Primary MRI analyses – excluded observed data obtained after switch to alternative MS medications. Missing data prior to alternative MS medications and visits after switch are imputed using the constant rate assumption.

^c^Odds ratio, 95% CI, and *p*-values for comparison between active and placebo groups at 2 years, based on ordinal logistic regression, adjusted for region and baseline number of Gd+ lesions.

BID = twice daily; DMF = dimethyl fumarate; Gd+ = gadolinium-enhancing; ITT = intent-to-treat; MS = multiple sclerosis; OR = odds ratio; TID = three times daily.

## Figure e-2

MRI outcomes in pre-specified patient subpopulations

Reductions at both doses of delayed-release DMF in T2 lesion number (A, B) and Gd+ lesion number (C, D) in pre-specified subpopulations based on baseline demographic and disease characteristics, over the 2 years of the study.


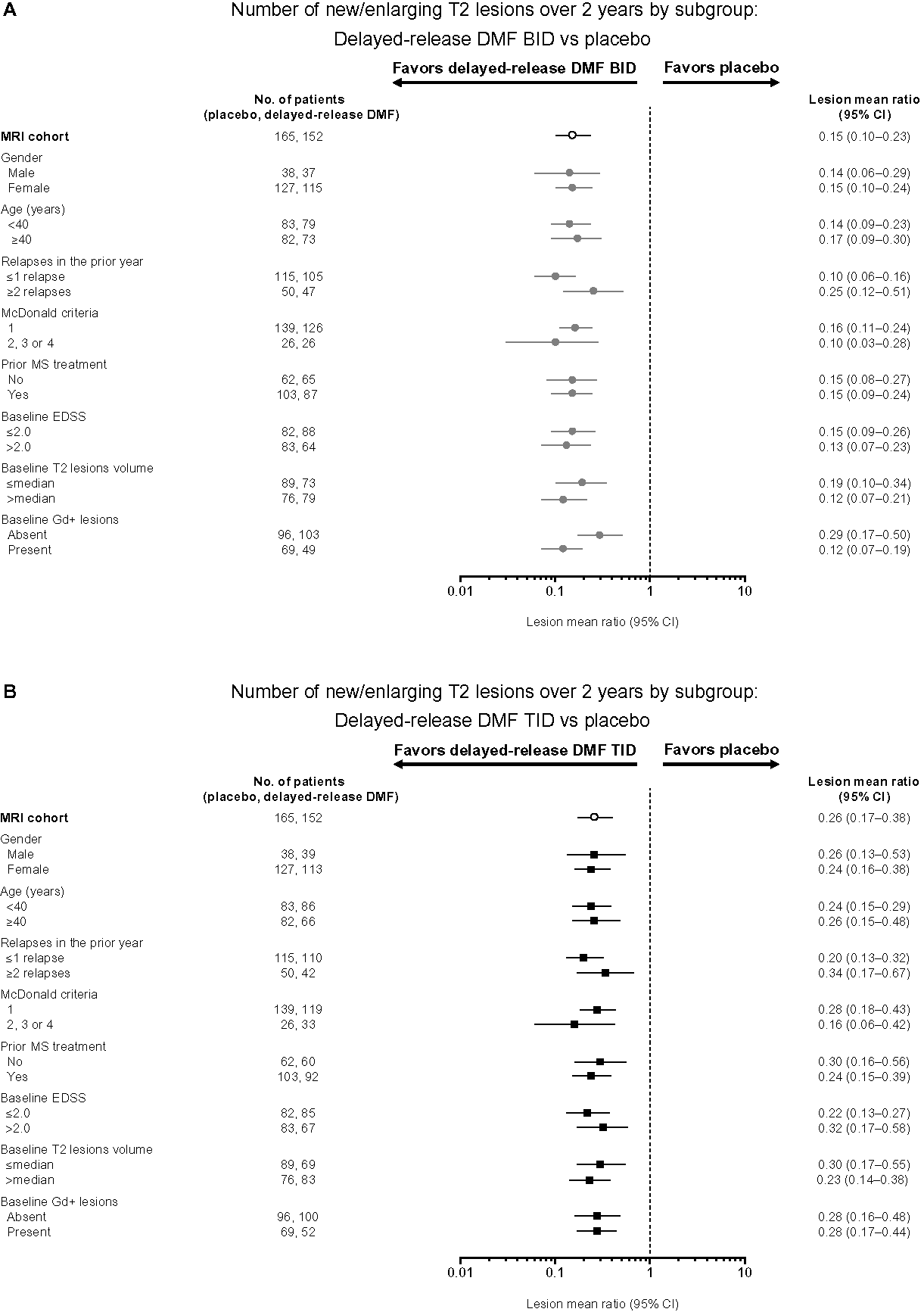


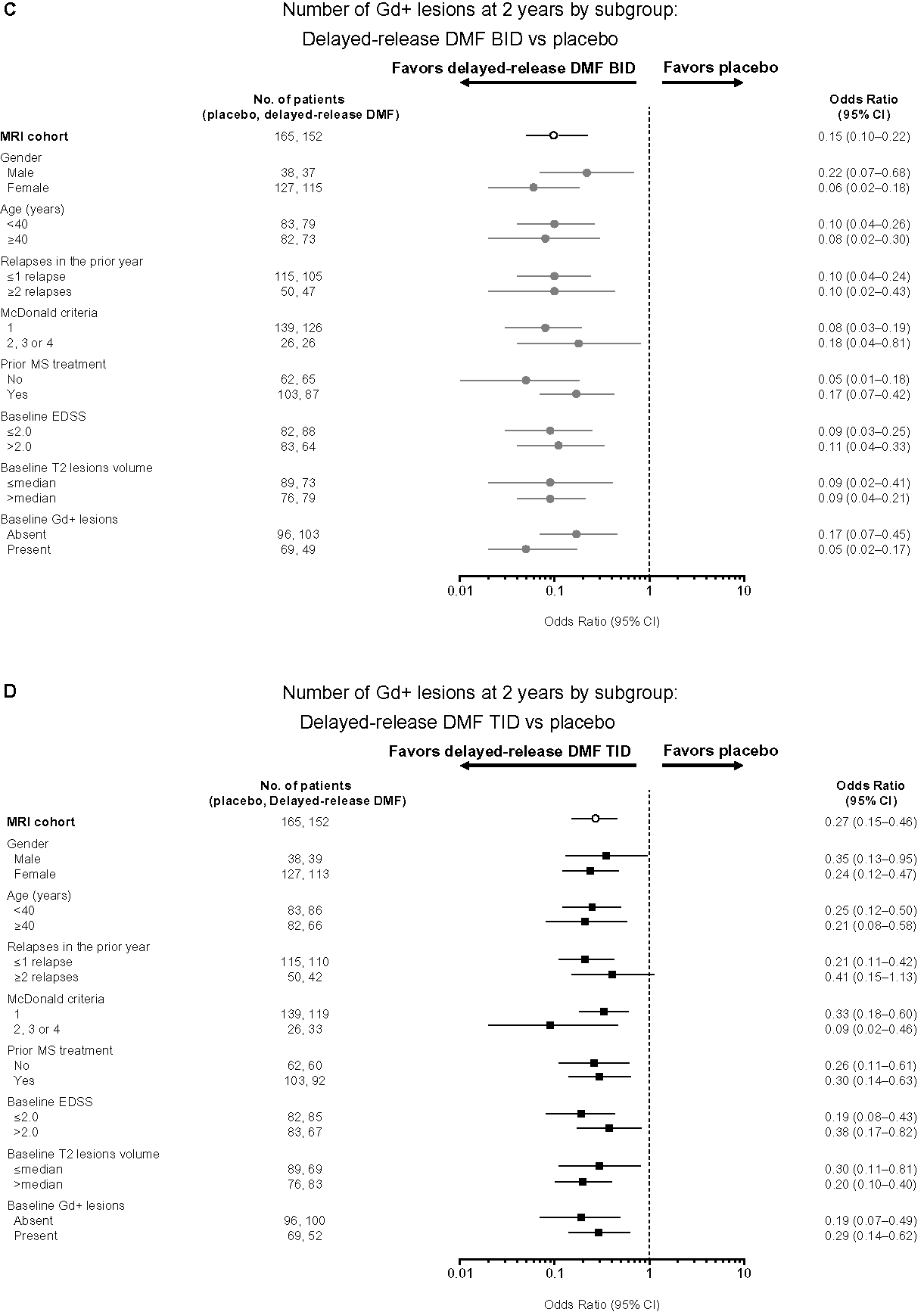


BID = twice daily; DMF = dimethyl fumarate; EDSS = Expanded Disability Status Scale; Gd+ = gadolinium-enhancing; MS = multiple sclerosis; OR = odds ratio; TID = three times daily.
