# Supplementary figures and images for: Effects of delayed-release dimethyl fumarate on MRI measures in the Phase 3 DEFINE study
Source: J Neurol. 2014 Jul 3;261(9):1794–802. doi: 10.1007/s00415-014-7412-x (PMC4155185; doi:10.1007/s00415-014-7412-x)

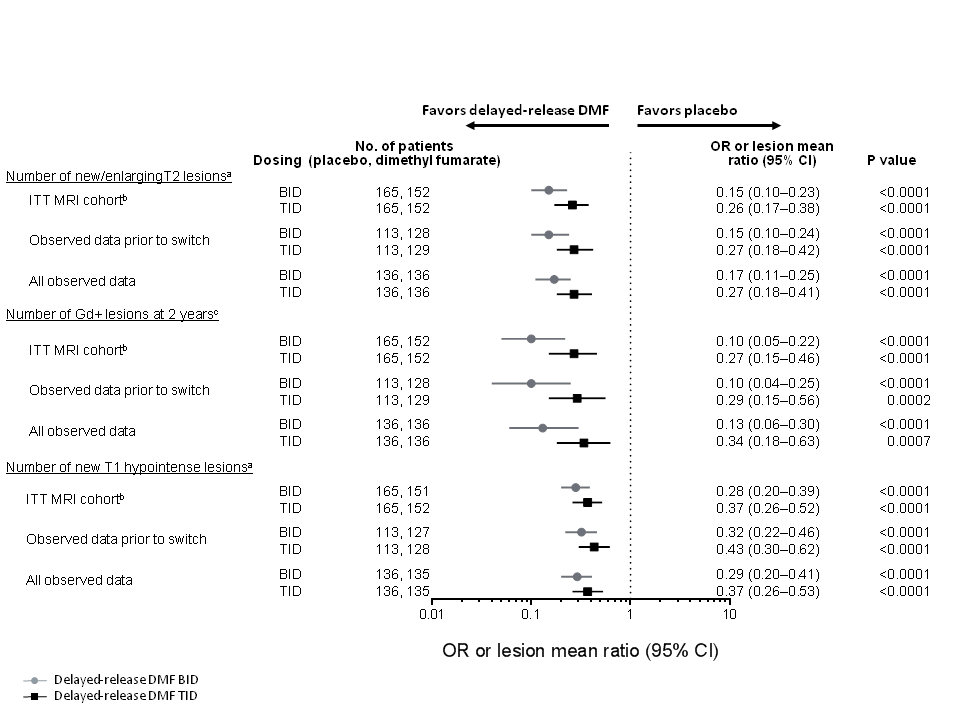

Supplement: Supplementary file 2 — Supplementary material 2 (TIFF 78 kb) [file 415_2014_7412_MOESM2_ESM.tif]

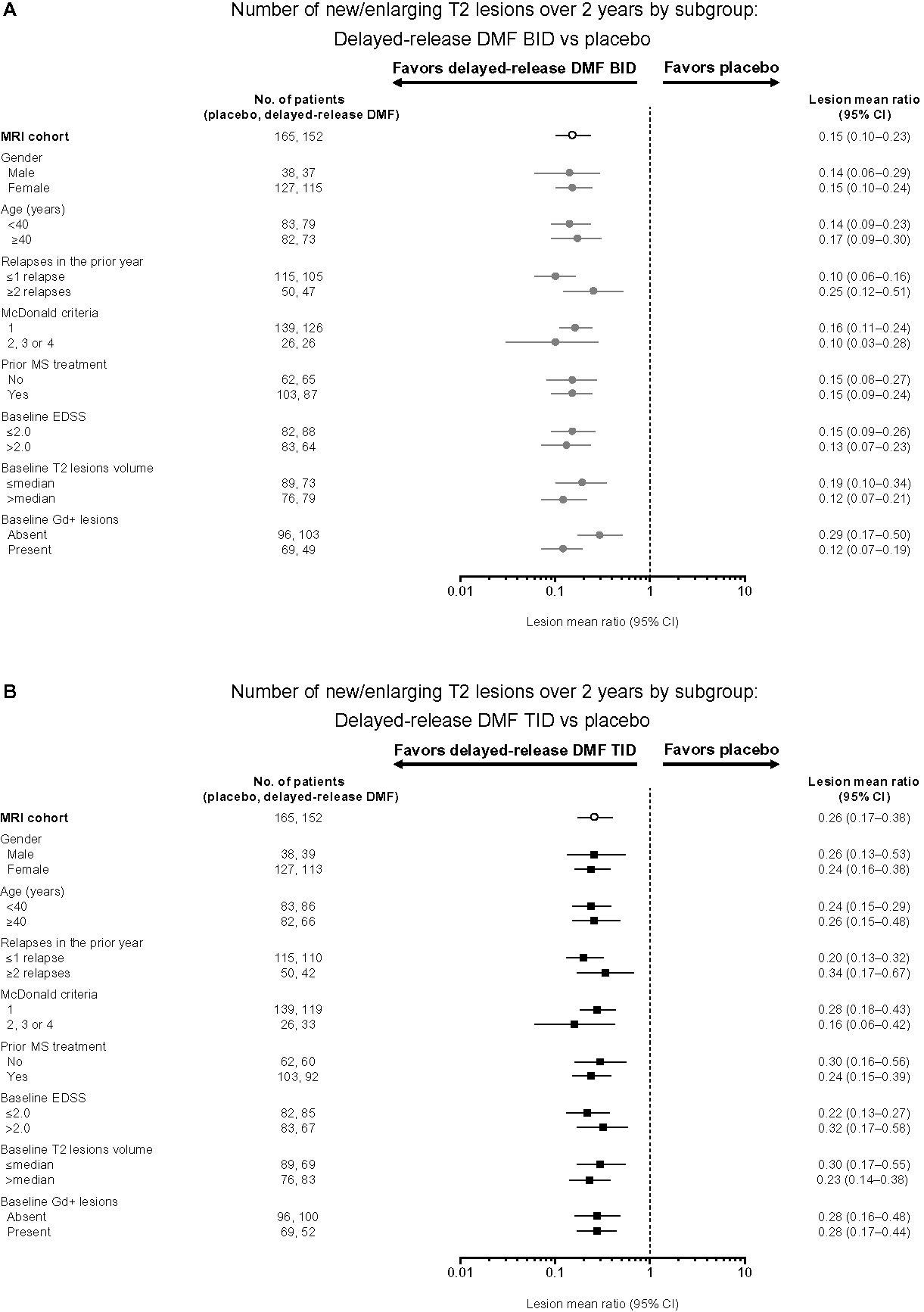

Supplement: Supplementary file 3 — Supplementary material 3 (TIFF 8570 kb) [file 415_2014_7412_MOESM3_ESM.tif]

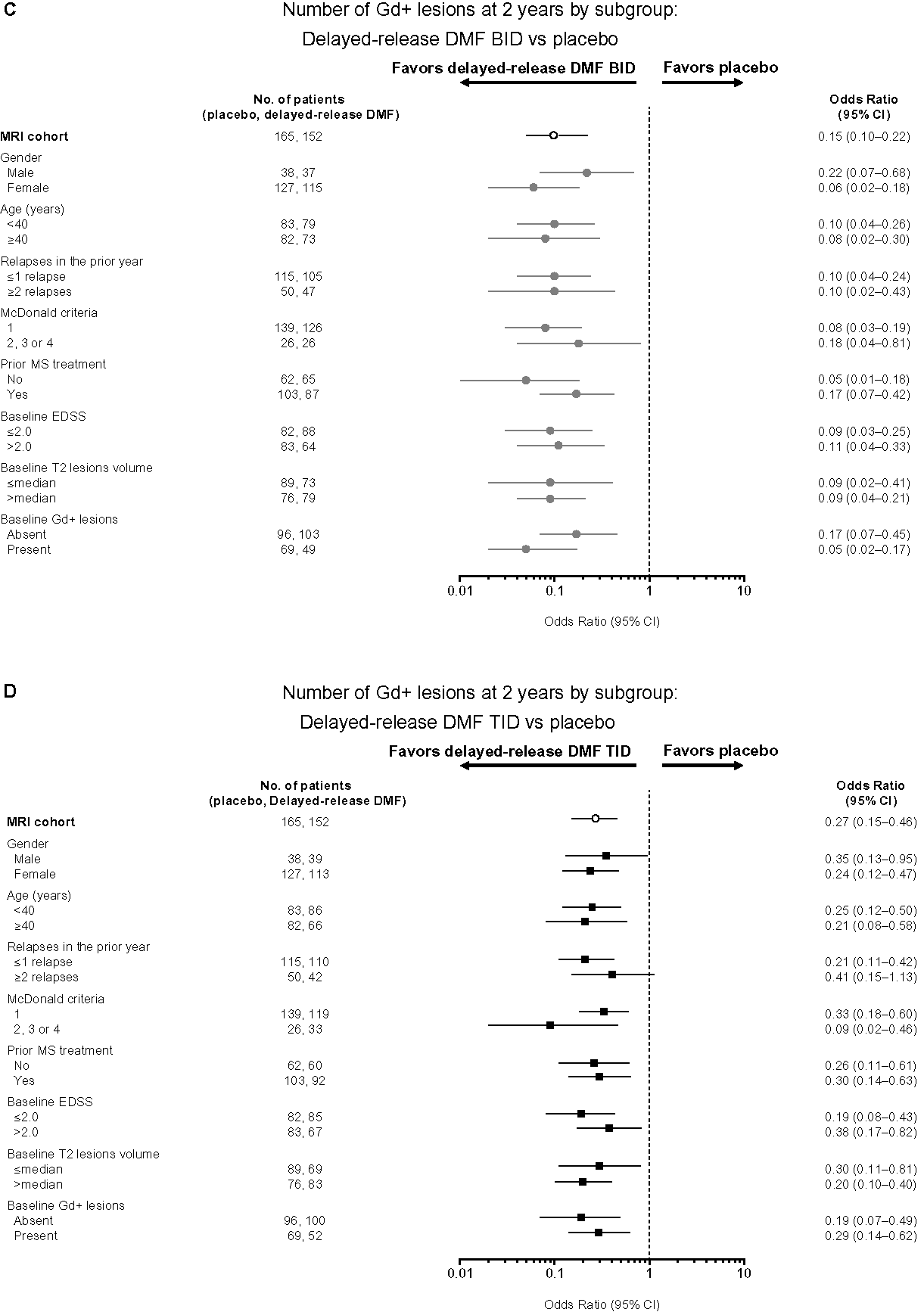

Supplement: Supplementary file 4 — Supplementary material 4 (TIFF 8591 kb) [file 415_2014_7412_MOESM4_ESM.tif]
